# Supplementary figures and images for: Mindfulness Teacher Trainees’ Experiences (MTTE): An investigation of intense experiences in mindfulness-based interventions
Source: PLoS One. 2024 Apr 5;19(4):e0301593. doi: 10.1371/journal.pone.0301593 (PMC10997133; doi:10.1371/journal.pone.0301593)

**Figure S1. Hierarchical node tree.**


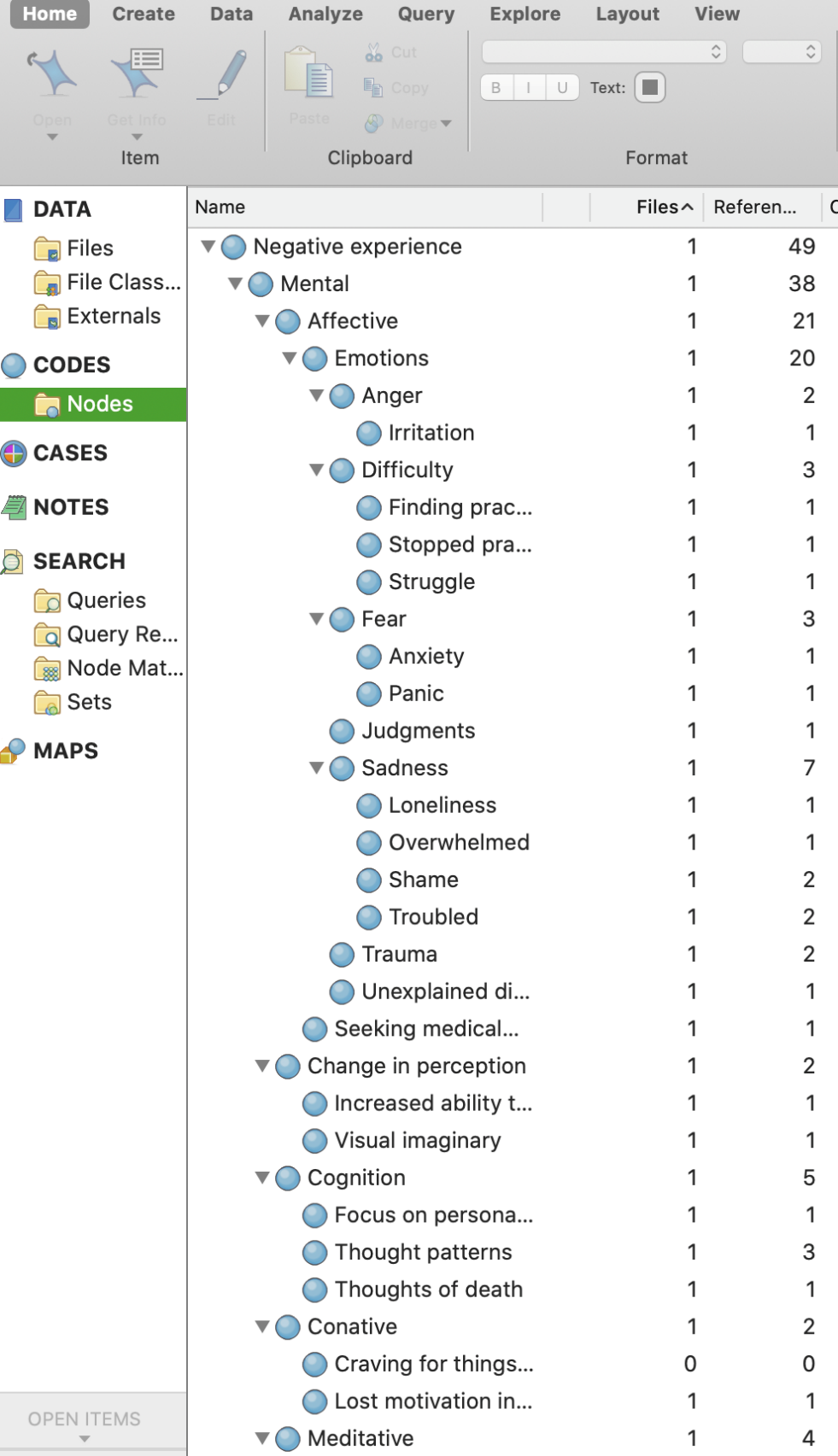


Figure S1. Hierarchical node tree.

Supplement: S1 Fig — (DOCX) [file pone.0301593.s001.docx]
